# Relationship Between White Matter Integrity and Plasma Leptin Levels in Drug-Naïve and Medicated Patients With Major Depressive Disorder

**DOI:** 10.3389/fnins.2019.00707

**Published:** 2019-07-12

**Authors:** Abdulrahman A. A. Amer, Yue Zhu, Shengnan Wei, Ran Zhang, Yang Wang, Jia Duan, Xiaowei Jiang, Yanqing Tang, Fei Wang

**Affiliations:** ^1^Department of Psychiatry, First Affiliated Hospital, China Medical University, Shenyang, China; ^2^Brain Function Research Section, First Affiliated Hospital, China Medical University, Shenyang, China; ^3^Department of Radiology, First Affiliated Hospital, China Medical University, Shenyang, China; ^4^Department of Geriatric Medicine, First Affiliated Hospital, China Medical University, Shenyang, China; ^5^Department of Psychiatry, Yale School of Medicine, New Haven, CT, United States

**Keywords:** major depressive disorder, white matter, leptin, DTI, thalamus

## Abstract

Many previous studies have noticed obvious alterations in different white matter tracts among patients with major depressive disorder (MDD). Growing evidence also strongly suggest a role of leptin in the pathogenesis of MDD, but with conflicting results of leptin levels. However, no previous studies have examined the relationship between leptin and white matter integrity of patients with MDD. Therefore, we aimed in this study to investigate the relationship between white matter alterations and plasma leptin levels in both drug-naïve and medicated MDD patients. We measured plasma leptin levels and white matter integrity using diffusion tensor imaging (DTI) and voxel-based analysis (VBA) in 140 participants (40 drug-naïve MDD patients; 40 medicated MDD patients; 60 healthy controls) aged between 18 and 49 years old. A significant reduced fractional anisotropy (FA) value in the dorsomedial thalamus was found for both drug-naïve and medicated MDD patients compared to the healthy non-depressed participants (*p* < 0.01, corrected). In addition, leptin levels were significantly higher in the drug-naïve MDD patients and were negatively correlated with the detected white matter alteration. Our results suggest that the elevated plasma leptin levels in the drug-naïve MDD group might be associated with the changes of the white matter integrity in the dorsomedial thalamus region.

## Introduction

White matter tracts are key components of a big complicated network that participates in somehow directly or indirectly in the course of major depressive disorder (MDD). Many studies have noticed this relation and discovered various anatomical and functional abnormalities of the white matter fibers which were associated to the severity and relieve of the depressive symptoms (Bessette et al., 2014; de Diego-Adelino et al., 2014).

One of the best ways to study the abnormalities of the white matter is to use the diffusion tensor imaging (DTI), a newly developed MRI technique, which can be used to characterize water movement in white matter fibers *in vivo*. Fractional anisotropy (FA) is a DTI-derived diffusivity measure which can reflect the directionality of water diffusion and is used to indicate the location and strength of white matter fibers.

In many DTI studies, FA values were found to be lower for patients with MDD compared to healthy individuals in different white matter regions such as the thalamus, frontal lobe, uncinate fasciculus, corpus callosum, cerebellum, parietal lobe, and superior longitudinal fasciculus (Wu et al., 2011; Bessette et al., 2014; Xia et al., 2018).

Theories of the causes of MDD have not yet been unified and pathophysiology of the disease is still partly understood.

The strong relationship found between obesity and depression led the way to explore the potential role of fat tissue in the cause of MDD through its production of leptin hormone (de Wit et al., 2010; Cao et al., 2018). Leptin is a peptide hormone predominantly produced by fat cells and participates in the regulation of weight and appetite which their changes are main symptoms of MDD. It is thought that the relationship between leptin and problematic eating behaviors and changes in appetite may be an important risk factor for weight gain in patients with MDD (Mills et al., 2018).

Studies of the relationship between leptin and major depression are gradually increasing. Recently, it is strongly suggested that leptin dysregulation is specifically associated with MDD (Milaneschi et al., 2017). However, conflicting results of leptin levels in depressed patients were reported, some reported that depressive symptoms were significantly correlated to the elevated plasma leptin levels (Antonijevic et al., 1998; Takekawa et al., 2019), lower and similar concentrations of leptin levels compared to healthy individuals were also reported (Deuschle et al., 1996; Jow et al., 2006; Hryhorczuk et al., 2013).

Blood leptin can be transported into the cerebrospinal fluid then into the brain across the blood brain barrier through its different forms’ receptor (ObR, a-f) (Ge et al., 2013). Many studies demonstrated a potential effect of leptin on the brain directly through its receptors distributed in the hypothalamus, it is the key adipokine that mediates the fat tissue-brain communication to regulate food intake and maintain energy balance (Jequier, 2002).

Some evidences had also implicated additional functions of leptin on regions outside the hypothalamus, such as the hippocampus which is critically involved in learning and memory processes (Harvey et al., 2006). Other regions receiving functions of leptin include cortex, thalamus, striatum, midbrain and other regions of the central nervous system with different regional thresholds for optimal function (Banks et al., 2000). However, the hypothalamus region is confirmed to uptake the highest volume of leptin (Faouzi et al., 2007).

Previous studies also showed that circulating blood leptin is not only derived from fat tissue, but also from the brain (Ahima et al., 1999; Ur et al., 2002). Therefore, it is thought that leptin has a direct neurodevelopmental action via its widely distributed receptors expressed in different brain regions which participate in learning, memory and the regulation of mood and emotions (Elmquist et al., 1997; Bouret, 2010).

There is an obvious lake of studies regarding the correlation between dysregulation of leptin levels and brain white matter abnormalities. Pannacciulli et al. (2007) studied the relation between plasma leptin concentrations and human brain structure in a group of lean and obese normal participants and did not find any positive or negative associations between plasma leptin and the volume of the white matter. However, it is widely noticed that obesity patients are associated with abnormally increase of leptin levels. This increase is believed to be cause by the reduced ability of leptin to suppress appetite and weight gain, hence, developing leptin resistance (Maffei et al., 1995; Zhou and Rui, 2013).

Obesity was found to be also associated with reduced white matter integrity, particularly in the genu, splenium, and fornix, suggesting a possible role for adiposity in white matter dysfunction and the associated cognitive deficits (Karlsson et al., 2013), however, leptin level was not evaluated in this study.

Despite growing evidences for white matter and leptin level abnormalities in MDD patients, there are no published studies examining their relationship.

We hypothesized that high leptin levels may alter white matter integrity in MDD patients, and reduced leptin levels may improve the disease.

Therefore, the aim of this study was to investigate the relationship between white matter alterations and plasma leptin levels in both drug-naïve and medicated MDD patients using voxel-based analysis (VBA).

## Materials and Methods

### Participants

Our study had included 140 participants (40 drug-naïve MDD patients; 40 medicated MDD patients; 60 healthy controls) aged between 18 and 49 years old and were recruited from the same site at the outpatient department of psychiatry, the First Affiliated Hospital of China Medical University, Shenyang, China. The recruitment interval was from February 2013 to July 2017.

Drug-naïve MDD patients was chosen as they have never used any kind of anti-depressants and medicated MDD patients as they have the history of using anti-depressants. Remitted MDD patients were excluded from this study. The healthy controlling individuals were matched for age, gender and education and were recruited by advertisement. A written informed consent was obtained from all participants prior to the study.

The diagnosis of MDD was made by two trained psychiatrists according to the guidelines given in the Structured Clinical Interview for Diagnostic and Statistical Manual of Mental Disorders, 4th Edition (DSM-IV) (SCID). All healthy controls were screened using the SCID Non-Patient Edition to confirm absence of DSM-IV Axis I and to exclude any psychiatric disorders.

Participants with diabetes, hypertension, vascular and infectious disorders and other major neurological or medical comorbidities, alcohol or drug abuse, head trauma, and MRI contraindications were excluded from the study.

The 17-item Hamilton Depression Rating Scale (HAMD-17) and the Hamilton anxiety Scale (HAMA) were used to assess the symptoms of all participants. Obesity was tested by body mass index (BMI) (BMI ≥ 30) (calculated as weight in kilograms divided by height in meters squared).

The study was approved by the Institutional Review Board of the China Medical University and was performed according to the principles of the Declaration of Helsinki.

### Image Acquisition and Analysis

MRI data were acquired using a 3.0T MR scanner General Electric Sigma system at the First Affiliated Hospital of China Medical University, Shenyang, China. A standard head coil was used for radiofrequency transmission and reception of the nuclear magnetic resonance signal. Foam pads were used to minimize head motion.

Diffusion tensor imaging data were acquired using a spin-echo planar imaging sequence. The diffusion sensitizing gradients were applied along 25 non-collinear directions (*b* = 1000 s/mm^2^), together with an axial acquisition without diffusion weighting (*b* = 0). Scan parameters were repetition time (TR) = 17 000 ms; echo time (TE) = 85.4 ms; image matrix = 120 × 120; field of view (FOV) = 24 cm × 24 cm; 65 contiguous slices of 2 mm and no gap.

### DTI Data Analysis

Standard and approved procedures were used to analyze DTI data (Ashburner and Friston, 2000). For this purpose, PANDA software (Pipeline for Analyzing brain Diffusion images^1^) were used.

Images of the diffusion metrics were transformed from native space to a standard Montreal Neurological Institute (MNI) using spatial normalization with voxel size of 2 mm^3^.

The fractional anisotropy (FA) value was calculated using default program parameters on a voxel-by-voxel basis.

Specifically, the FA image of each subject was non-linearly registered to the FMRIB58_FA template. FA images were smoothed with a 6-mm full width at half maximum Gaussian filter.

### Measurement of Plasma Leptin Levels

Five milliliters venous blood samples were collected from all participants between the time 10:00 AM and 3:00 PM and were centrifuged at 2,000 rpm for 10 min then stored at −80°C for leptin measurements. Ethylenediamine tetra acetic acid (EDTA) was used as an anticoagulant.

Levels of leptin were measured using the Human Premixed Multi-Analyte Kit (R&D Systems, Inc., Minneapolis, MN, United States) with the Human Magnetic Luminex Assay (Leptin [BR51]). Samples were magnetically labeled using a human, magnetic, premixed, microparticle cocktail of antibodies (Kit Lot Number L120614). All participants underwent the MRI scan within 24 h after collecting blood samples.

### Statistics

For assessing differences between groups, one-way analysis of variance (ANOVA), two-sample *t*-tests, and the chi-square test were performed using IBM SPSS Statistics for Windows, Version 23.0.

The ANOVA test was also used to assess differences among the three groups in the white matter of the brain. The significance was set on voxel *p* < 0.01 corrected using Gaussian Random Field (GRF) method. *Post hoc* analyses were conducted using Bonferroni tests. For all statistical tests, *p* < 0.05 was considered statistically significant.

Partial correlation test controlling for age was performed to assess the relation between leptin and FA values.

## Results

### Demographic and Clinical Data

No significant differences were noticed between groups in the recorded demographic data including age, gender, education and BMI. No significant difference in the disease duration between drug-naïve and medicated MDD patients (*p* = 0.146). As expected, three groups were significantly different in depression and anxiety symptoms. The demographic and clinical data of all different groups are presented in Table 1.

**TABLE 1:** Demographic and clinical characteristics of all groups.

**Characteristics**		**Drug-naïve *N* = 40**	**Medicated *N* = 40**	**HC *N* = 60**	**Statistics**	***P*-value**
**Demographic characteristics**						
Gender	Male	12 (30%)	10 (25%)	28 (46.7%)	5.704^a^	0.058
	Female	28 (70%)	30 (75%)	32 (53.3%)		
Age		27.0 (8.2)	30.1 (6.9)	28.4 (6.4)	1.975^b^	0.143
Education (years)		13.3 (2.8)	13.5 (3.2)	14.6 (3.1)	2.782^b^	0.065
BMI		21.8 (3.7)	21.2 (3.6)	22.2 (4.1)	0.824^b^	0.441
**Clinical characteristics**						
Disease duration (months)		19.6 (28.2)	37.2 (65.6)	N/A	−1.470^c^	0.146
HAMA (*N* = 38/39/60)		15.3 (11.8)	14.3 (11.0)	1.3 (2.6)	40.764^b^	0.000
HAMD (*N* = 40/39/60)		17.3 (9.5)	15.3 (9.4)	1.1 (2.0)	76.740^b^	0.000
**Examinations**						
Leptin pg/ml (N = 19/32/60)		9816.9 (7399.1)	5047.7 (4012.1)	6210.4 (4662.2)	5.495^b^	0.005
DTI FA value (N = 39/40/60)		0.268 (0.026)	0.275 (0.015)	0.287 (0.027)	8.490^b^	0.000

*Number N, percentage (%), mean and standard deviation (SD) are listed in the table. HC, healthy controls; HAMA, Hamilton anxiety Scale; HAMD, Hamilton Depression Scale; ^*a*^χ^2^-test; ^*b*^one-way ANOVA test; ^*c*^two-sample *t*-test.*

### Comparison of DTI Measures

**FIGURE 1:**
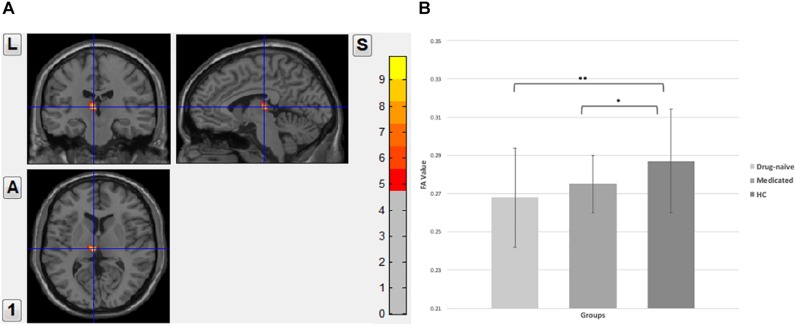
**(A)** Fractional anisotropy value comparison between the study groups showing abnormalities in the left thalamus, corrected with voxel *p* < 0.01. **(B)** *Post hoc* comparison showing FA values differences between each pair group, ^∗∗^significant at *p* < 0.001, ^*^significant at *p* < 0.05.

The applied one-way analysis of variance (ANOVA) detected one cluster in the white matter at *p* < 0.01 showing significant different between the three groups located in the left thalamus, particularly in the dorsomedial thalamus (MNI coordinates for the maximal point of difference: *x* = −6 mm, *y* = −20 mm, *z* = 8 mm, number of voxels 99 and cluster size 73, *F* = 7.850, *p* < 0.01, corrected). *Post hoc* comparison showed significantly lower FA value in the drug-naïve MDD patients when compared to the healthy control group (*p* < 0.001). Medicated MDD patients had also significantly reduced FA value when compared to the HC group (*p* < 0.05). No significant difference was detected between Drug-naïve and medicated MDD patients (Figure 1).

### Comparison of Plasma Leptin Levels

Significant differences were observed between the three groups (*F* = 5.544, *P* = 0.005). *Post hoc* analyses showed that plasma Leptin levels were significantly higher in the drug-naïve MDD patients compared to the medicated patients and healthy controls. Mean of leptin levels were slightly reduced in the medicated MDD patients but were not statistically significant in *post hoc* analyses when compared to the healthy controls. In all study groups, no correlation was found between plasma leptin levels and BMI, age or either gender (*P* > 0.05). However, because of some missing data, the collected number of leptin samples in MDD patients were less than the total enrolled participated number.

### Correlation Between Plasma Leptin Levels and FA Values

**FIGURE 2:**
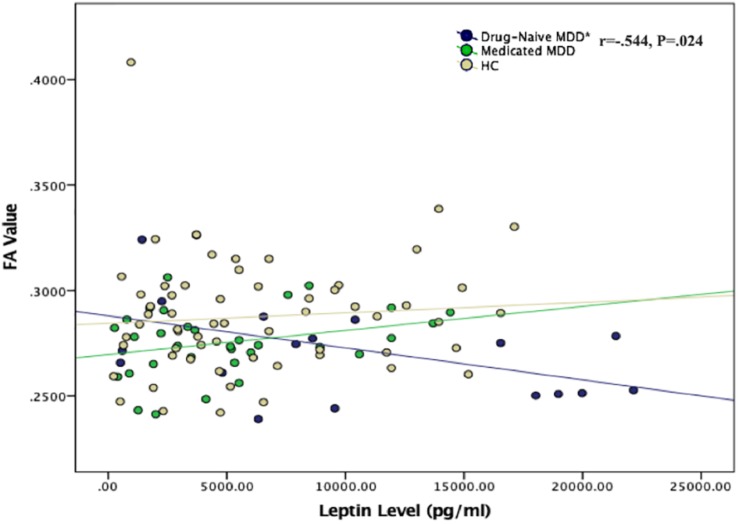
Scatter plot of the correlation between plasma leptin levels and FA values in all study groups. ^*^Negative correlation was found between plasma leptin level and FA value in the left thalamus region in drug-naïve MDD group (*P* = 0.024). No correlation was found for the medicated MDD and healthy control groups.

Partial correlation test between two groups separately found that high levels of plasma leptin were significantly associated with reduced FA values in drug-naïve MDD patients when controlling for age (Figure 2). No correlation was found between leptin and FA values in medicated MDD patients or either the healthy controls.

## Discussion

In the present study, MDD patients had an obvious alteration of the white matter integrity particularly in the thalamus region, a significant reduced fractional anisotropy (FA) value in the dorsomedial thalamus was found for both drug-naïve and medicated MDD patients compared to the healthy non-depressed participants. In addition, leptin levels were significantly higher in the drug-naïve MDD patients and were negatively correlated with the detected white matter alteration.

This study provides the first evidence of the relationship between plasma leptin levels and the abnormalities of the white matter among patients with MDD.

Diffusion tensor derived directional water diffusivities are potential markers which can detect and differentiate axon and myelin injury, therefore, reduction of the fractional anisotropy (FA) values is thought to be reflecting the reduction of the white matter organization, reduction of axonal density and/or reduction of myelination (Beaulieu, 2002; Basser and Pierpaoli, 2011). Although the exact cause of the FA values reduction in the thalamus region in MDD patients remain poorly understood, however, the thalamus was proved previously to play a critical role in the pathophysiology of depression (Greicius et al., 2007).

Abnormalities of this region are expected because the thalamus is thought to serve as a critical center of integration of networks such as the frontal cortex and basal ganglia which underlie the ability to modulate behaviors, mediate motivation and emotional drive, and participate in planning and cognition behaviors (Haber and Calzavara, 2009; Sherman, 2014).

Many studies also observed the existence of specific connections between different regions of the thalamus and the limbic system suggesting an important role of the thalamus in the frontal-limbic dysregulation which is seen in depression (Taber et al., 2004). Loss of white matter integrity found in MDD particularly in the thalamic projection fibers, limbic system and frontal cortex also support the theory of limbic-dorsolateral prefrontal cortex-thalamic dysfunction in depression (Korgaonkar et al., 2011).

The specific area of the thalamus, the dorsomedial, is proved to integrate specific communications with the frontal lobes and involve in the regulation of cortical networks especially when the maintenance and temporal extension of persistent activity patterns in the frontal lobe areas are required, playing a critical role in higher cognitive functions together with the prefrontal cortex (PFC) and other brain regions (Mitchell and Chakraborty, 2013; Pergola et al., 2018).

In addition, it is also highlighted that the neural basis of memory and cognitive deficits are associated with the subgroupings of the dorsomedial thalamus (medial, central, and lateral) and their interconnected neural networks (Mitchell and Chakraborty, 2013).

Therefore, in case of major depression, the detected elevation in neuron number in the dorsomedial thalamus (Young et al., 2004), along with our detected damage of this region and the previous findings of the deficits in the tracts between the dorsomedial thalamus and other regions (Osoba et al., 2013), all these findings support the presence of both structural and functional abnormalities in dorsomedial thalamus region in MDD patients.

The high leptin levels found in our study were also supported by the results of several studies. Morris et al. (2012) found that leptin was significantly increased in depressed patients and was positively correlated with the severity of the disease, participants with moderate to severe depression had higher levels of leptin than those with mild or minimal to no depression. Gecici et al. (2005) and Milaneschi et al. (2017) found that leptin was significantly higher in patients with MDD but for only the atypical subtype (hyperphagia, increased weight, and leaden paralysis) compared to non-atypical patients, suggesting that leptin resistance may represent an underlying mechanism linking increase of weight with depression.

However, our study recorded a normal range of body mass index (BMI) in all study groups and did not find any correlation between BMI and leptin levels. This result was also supported by Antonijevic et al. (1998) study which showed that depression was associated with higher leptin levels but with lacking positive correlation between leptin and body weight.

In terms of the negative relationship that we found between plasma leptin levels and changes of the brain white matter in MDD patients, we suggest that high levels of plasma leptin in MDD patients may associate with the alternations of some specific regions of the white matter such as the thalamus which was significantly influenced in this study.

Since the brain is known to be the major site that mediates the function of leptin (Cohen et al., 2001), the negative relationship in this study may reflect a direct action of leptin on the white matter, or indirect action on other central-signaling molecules that causes damage to the brain. Previous findings have provided some evidences which can help explaining the possible mechanisms underlying this result.

Leptin was proved previously to be positively associated with the glucocorticoids, which are responsible for the hyperactivity of the hypothalamic-pituitary-adrenal axis (Newcomer et al., 1998), increasing during depression and may cause damage to the structure of brain (Liu et al., 2016). Elevated glucocorticoids are proposed to inhibit the proliferation of astrocytes and oligodendrocytes, which participate in the uptake, metabolism and recycling of glutamate and are responsible for myelinating the axons of white matter tracts (Rajkowska and Miguel-Hidalgo, 2007). Elevated cortisol levels in major depressed patients was shown to be correlated with the reduction of some specific white matter circuits including the thalamic radiation (Liu et al., 2016).

In addition, leptin was shown to be responsible for the inhibition of the Neuropeptide Y (NPY) neurons, which is known to have a specific anxiolytic and neuroprotective properties (Reichmann and Holzer, 2016), and is proposed to play a critical role in mediating chronic stress in patients with depression (Widerlov et al., 1988; Jimenez Vasquez et al., 2000).

However, leptin receptors was shown to be abundantly expressed in the thalamus region (Caron et al., 2010), supporting our speculation that alternations of the thalamus may occur under high plasma leptin levels in the MDD brain as shown in this study.

In the contrast, some studies demonstrated that leptin have antidepressant and anxiolytic effects and might be a potential therapeutic target for depression (Lu et al., 2006; Ge et al., 2018). Some studies have reported that leptin may modulate fear and anxiety behaviors through the reversal of conditioning−induced potentiation of thalamic input synapses onto the lateral amygdala (Wang et al., 2015).

Our results suggested that high leptin levels in MDD patients is associated with changes in the white matter and might injure the microstructures in some specific related white matter circuits. Thus, indicating a major role of leptin in the pathogenesis of MDD.

One of the advantages of our study is that we separately recruited both medicated and unmedicated MDD patients to additionally examine the effect of drug use on the changes of plasma leptin levels or white matter abnormalities.

In the present study, levels of plasma leptin were significantly lower in patients with psychotropic medication history compared to non-medicated patients and were in the normal range compared to the healthy non-depressed participants. The result may reflect medications’ effects on levels change of leptin, however, future longitudinal studies are needed to verify this relationship.

There is an obvious lake of studies comparing leptin levels among depressed patients with and without medication history. Kraus et al. (2001) recorded lower leptin levels and normal BMI for MDD patients compared to healthy controls, but this leptin reduction did not depend on whether or not the patients had been treated using psychotropic medication during the past 4 weeks. In contrast, Esel et al. (2005) found that leptin levels were higher both before and after the response to antidepressant treatment, with further elevation on the leptin levels after the improvement from depression with antidepressant treatment.

However, the statically significant differences in leptin levels and the non-statically variances in FA values that we found between drug-naïve and medicated MDD patients, and the negative correlation between leptin levels and only the drug-naïve MDD group indicate that the use of psychotropic drugs directly or indirectly reduces leptin levels which may positively improve the disease.

There are some limitations in the present study that should be considered when interpreting the results. First, we lacked information on the use of different types of antipsychotic drugs and exact duration of medication for the medicated MDD participants, and the association of the use of different antipsychotic drugs with the differences in white matter changes could not separately explained.

Participants were not fasting during the blood collection, fasting, and non-fasting comparison should be considered for future studies.

Our study performed the partial correlation controlling only for age which have been reported previously to have major effect on the brain. Future studies should consider controlling for other factors such as gender and BMI.

The age of enrollment in this study was designed without a strong basis, comparison of different age periods should be considered in future studies.

The effects of the disease duration, number of episodes and other potential variables such as economic level, lifestyle, dietary habits were not controlled in this study.

The relatively small sample size and missing of some data decreased the power of our results. More consistent demographic characteristics in a large sample size should be considered in future studies.

This study did not measure other metabolic factors that are reported to have relation with leptin such as cortisol and Insulin levels (Newcomer et al., 1998), future studies should consider the association between different metabolic factors and leptin in MDD patients.

## Conclusion

In conclusion, we have shown that fractional anisotropy value of the left dorsomedial thalamus was significantly decreased for both medicated and unmedicated patients with major depression disorder, while plasma leptin secretion was enhanced and significantly correlated with FA value for only the latter, indicating that the microstructural abnormality of the white matter found in MDD patients is associated with high leptin levels. White matter changes might be related to the high plasma leptin levels and medications might help to improve these changes. However, confirmation of this relationship should be further explored in future longitudinal studies.

Our results need to be confirmed by more comprehensive studies with detailed demographical and psychological data with clinical symptoms, metabolic factors and specific medication information to decipher the roles of leptin in the pathophysiology of depression and brain abnormalities.

## Data Availability

The datasets for this manuscript are not publicly available because the raw data supporting the conclusions of this manuscript will be made available by the authors, without undue reservation, to any qualified researcher. Requests to access the datasets should be directed to abdu_amer2005@hotmail.com.

## Ethics Statement

The study was approved by the Institutional Review Board of the China Medical University and was performed according to the principles of the Declaration of Helsinki. A written informed consent was obtained from all participants prior to the study.

## Author Contributions

AA and FW designed and wrote the manuscript. All authors participated in the collection, analysis, and interpretation of the data. All authors discussed the results, reviewed and approved the final version of the manuscript.

## Conflict of Interest Statement

The authors declare that the research was conducted in the absence of any commercial or financial relationships that could be construed as a potential conflict of interest.

## Footnotes

1 http://www.nitrc.org/projects/panda
